# 血管紧张素Ⅱ及其受体与恶性肿瘤关系的研究进展

**DOI:** 10.3779/j.issn.1009-3419.2016.09.10

**Published:** 2016-09-20

**Authors:** 鹿璐 孙, 健 史

**Affiliations:** 1 050000 石家庄，河北医科大学 Graduate Student of Hebei Medical University, Shijiazhuang 050000, China; 2 050000 石家庄，河北省肿瘤医院肿瘤内科 Department of Medical Oncology, Hebei Province Cancer Hospital, Shijiazhuang 050000, China

**Keywords:** 肿瘤, 血管紧张素Ⅱ, 血管紧张素Ⅱ1型受体, 血管紧张素Ⅱ2型受体, Tumor, Angiotensin Ⅱ, Angiotensin Ⅱ 1 receptor, Angiotensin Ⅱ 2 receptor

## Abstract

血管紧张素Ⅱ（angiotensin Ⅱ, AngⅡ）是由8个氨基酸组成的线性小肽，是肾素-血管紧张素系统（renin-angiotensinsystem, RS）主要的效应因子。AngⅡ主要的作用受体有两个，血管紧张素Ⅱ1型（angiotensin Ⅱ 1 receptor, AT1R）和AT2R分别是AngⅡ作用于靶细胞表面的特异性G蛋白偶联1型和2型受体。AngⅡ通过上述两种受体参与调节血管舒缩、水盐平衡、炎性反应、细胞增殖、细胞凋亡等生物学功能。近年来发现AngⅡ具有促进肿瘤细胞增殖、肿瘤血管形成并抑制肿瘤细胞分化的功能。这提示抑制AngⅡ的产生或阻断其作用有望成为治疗恶性肿瘤的一项新措施。本文就近年来关于AngⅡ及其受体与恶性肿瘤关系的研究进展作一综述。

## Abstract (en)

Angiotensin AngⅡ, a linear small peptide, which is composed of eight amino acids, is the main effectors of renin-angiotensin systen (Renin-angiotensin system, RAS). AngⅡ, a main biopolypeptide of the RAS, has important pathophysiologic in effects participating in cardiac hypertrophy, vascular cell proproliferation, inflammation and tissue remodeling through G-protein-coupled receptors. In recent years, Ang Ⅱ can promote tumor cell proliferation, tumor vessel formation and inhibit the differentiation of the tumor cells. This suggests that inhibit the production of AngⅡ or block its effect is expected to become a new measure for the treatment of malignant tumors. This article reviews the advances in research on the relationship between AngⅡ and its receptor and malignant tumor in recent years.

肾素-血管紧张素系统(renin-angiotensin system, RAS）是体内一个重要的神经-体液调节系统，其中血管紧张素Ⅱ（angiotensin Ⅱ, AngⅡ）是其主要的效应分子。近年来发现AngⅡ不仅存在于循环系统中，而且在局部组织，特别是肿瘤组织中表达明显，对于肿瘤的发生、发展、转移起到了重要作用。肺癌是目前临床上最为常见的恶性肿瘤之一，肺癌发病率每年增加。统计报告^[1]^认为美国已有412, 230例肺癌患者，每年新增肺癌患者将有226, 160例。肺癌发病隐匿不易察觉，目前特异性较高的早期诊断方法缺少，56%的肺癌患者就诊时就被确诊是恶性转移瘤，只有15%的肺癌患者就诊时能够确诊为原位癌。近年来，肺癌被认为是许多遗传因素和环境因素之间的相互作用所造成的一个复杂的多因素疾病^[2, 3]^。国内外多篇报道^[4, 5]^认为AngⅡ及血管紧张素Ⅱ1型（angiotensin Ⅱ 1 receptor, AT1R）的过度表达与肿瘤细胞的恶性生物学行为密切相关，但鲜见其与肺癌发生、发展关系的报道。以下将通过对AngⅡ及其受体、及两者与肿瘤之间关系的描述，分析其与肺癌之间的关系。

## 1 AngⅡ概述

### 1.1 来源

AngⅡ生成的两条途径分别是经典途径和旁路途径。两条途径均由血管紧张素原经一系列的酶促反应生成。血管紧张素原是由肝脏产生的一种球蛋白。经典途径是由肾素裂解血管紧张素原启动的。肾脏缺血是刺激肾素释放、激活RAS的最主要因素。血管紧张素原在肾素作用下，其氨基端第10位亮氨酸和第11位撷氨酸之间的肽链断裂生成10肽的AngⅠ。血管紧张素转换酶（angiotensin converting enzyme, ACE）是一种锌金属蛋白酶，主要存在于肺血管内皮细胞，也可见于子宫、胎盘、心脏、血管、肾、大脑、肾上腺皮质等大多数组织^[6]^。AngⅡ产生的旁路途径，即非肾素转化途径。ACE将AngⅠ转换为AngⅡ（血管收缩剂）和降解缓激肽（血管舒张药），在体内调节血压和心血管平衡^[7, 8]^。大约90%的AngⅠ转换为AngⅡ发生在肺部。除肾素之外，血管紧张素酶及组织蛋白酶亦可将血管紧张素原转化为AngⅠ，而胰蛋白酶、糜酶、组织蛋白酶G也能促使AngⅠ转化为AngⅡ。组织纤溶蛋白激活因子、组织蛋白酶G、血管紧张素酶等还能将血管紧张素原直接转化为AngⅡ。

### 1.2 作用

AngⅡ是RAS系统中最主要的生物效应因子，除了在血浆中存在的AngⅡ，已发现在各种组织局部也存在有功能的RAS，导致局部AngⅡ的产生。所以，可能在组织局部存在着旁分泌或者自分泌功能。在人体内，肾上腺皮质球状带细胞、脑部和血管平滑肌的某些部位、肾脏及心脏的一些细胞上存在血管紧张素受体。AngⅡ与其受体相结合，作用于血管平滑肌，收缩全身微动脉，从而升高动脉血压。并且，AngⅡ是目前发现的收缩血管能力最强的活性物质之一。以往，AngⅡ仅被看作是缩血管多肽，但有研究^[9]^发现不仅对于血管平滑肌细胞而且对于某些癌细胞系都是强有力的促生长因子。AngⅡ调节血管的收缩、细胞的生长、凋亡和分化，参与细胞的迁移、细胞外基质的形成、炎症的进展，另外还刺激几种生长因子的产生，如血小板源性生长因子、表皮生长因子、胰岛素样生长因子-1、基础纤维母细胞生长因子、血管内皮生长因子和血小板活化因子^[10-11]^。AngⅡ可激活第二信使系统，快速产生三磷酸肌醇和二酯酰甘油，并能使蛋白激酶C活化，继而增加细胞内钙水平，促进与生长有关的因子转录，以促进细胞的生长增殖^[12]^；同时，AngⅡ还可通过刺激癌细胞ERK1、ERK2、NF-κB、PKC和AP1信号途径提高血管内皮生长因子（vascular endothelial growth factor, VEGF）的表达，从而参与肿瘤的炎症反应、血管增生和细胞增殖^[13]^。

## 2 AngⅡ受体概述

AngⅡ受体有AT1R、AT2R、AT3R、AT4R四种亚型，其中起主要作用的是AT1R和AT2R，但两者的同源性仅24%-34%，且不同物种同源性有所不同。AT1R和AT2R的分布不同，AT1R在几乎所有的组织器官中广泛分布，如血管壁、脑、肝、肺、肾、肾上腺及垂体等。AT2R则分布局限，大部分在胎儿组织中，出生以后表达较少，甚至消失，只有当机体受到损伤，需要重新修复时才再次表达。且AT1R和AT2R之间的作用是相互拮抗的，在多种组织和器官中，AngⅡ可通过AT1R发挥其类似一种细胞因子的作用，促进细胞的生长和增殖；而通过AT2R则诱导如内皮细胞、成纤维细胞和平滑肌细胞等多种细胞的凋亡。因此，多年来大都认为AT1R和AT2R在调节细胞生长增殖方面的生物功能是对立的。由此推测，具有自分泌和旁分泌作用的AngⅡ可通过AT1R和AT2R两个受体来调节细胞的生长和凋亡；而AT1R介导了AngⅡ的绝大部分功能。AT1R能够促进肿瘤的生长以及肿瘤血管的生成^[13]^，AngⅡ正是通过激活AT1R促使了肿瘤的生长以及转移^[14]^。

## 3 AngⅡ及其受体与恶性肿瘤的关系

### 3.1 AngⅡ及AT1R在肿瘤组织中的表达

近年来有报道称，AngⅡ及其AT1R在许多癌组织中表达量增高，这些肿瘤包括前列腺癌、皮肤癌、子宫颈癌、子宫内膜癌、绒毛膜癌、卵巢癌、乳腺癌、胰腺癌、肺癌、肝癌、脑肿瘤、皮肤癌、咽喉癌等；而在癌旁组织中，AngⅡ及其AT1R表现出低表达。并且其表达与临床分期有关，分期越晚，组织分化越差，则表达越强^[15]^。在肿瘤组织中，AngⅡ主要通过与位于靶细胞表面的AT1R结合，从而促进肿瘤的生长转移。体内研究证明，应用AT1R的阻断剂可使肿瘤相关的VEGF减少，从而抑制血管生成，继而拮抗肿瘤生长^[16, 17]^。

### 3.2 AngⅡ及AT1R与肾癌

Dolley-Hitze等^[18]^通过免疫组织化学法、蛋白质印迹法和实时定量聚合酶链反应法检测发现，AT1R在肾透明细胞癌中的表达明显高于在正常组织中的表达。Tanabe等^[19]^在检测近肾小球细胞肿瘤组织中的AT1R和AT2R mRNA的水平时发现，AT1R的表达量增高，而AT2R在正常和肿瘤细胞中检测均未存在，猜想AngⅡ对体内肾素分泌的短循环反馈抑制，与AT1R在近肾小球肿瘤细胞中的表达水平有关。

### 3.3 AngⅡ及AT1R与前列腺癌

AT1R在正常前列腺、前列腺增生以及前列腺癌组织的间质中广泛存在，尤其在尿道周边区域的平滑肌基质中密集分布。AT1R在前列腺癌组织中表达量要高于正常前列腺细胞的表达量，且AT1R mRNA表达量亦增高。Kubota等^[20]^发现与正常前列腺组织相比，AT1R在前列腺癌组织中表达量增高，并推测AngⅡ及其AT1R与雄激素非依赖性前列腺癌的增长有关。

### 3.4 AngⅡ及AT2R与肿瘤

近年来人们更加深入的了解AngⅡ受体与肿瘤关系，而AT2R与许多肿瘤之间的关系也愈发浮出水面。有研究^[21]^表明，用激活剂激活的AT2R，能够对小鼠胰腺癌细胞产生抑制作用。AT2R可抑制小鼠结肠癌细胞的增殖，诱导细胞凋亡^[22]^；并且近期的研究^[23, 24]^中发现，在前列腺癌和肺癌中高表达的AT2R，不仅能抑制细胞的生长，还能诱导细胞的凋亡。这再一次证明了AT2R可作为肿瘤基因治疗的新靶点。有研究^[25]^发现，AT2R发挥促肿瘤细胞凋亡的作用不是通过传统的AngⅡ激活的G蛋白偶联受体通路，而是通过非配体-受体依赖，也就是说AT2R促进细胞凋亡无需AngⅡ的刺激，关键是AT2R自身的表达水平，这提示AT2R本身可能是存在于细胞内的一种凋亡诱导因子。由此可见，AngⅡ及其受体与恶性肿瘤的发生、发展之间有着密不可分的关系。

## 4 AngⅡ及其受体与肺癌的关系

### 4.1 AngⅡ与肺癌发生的机制

在过去的十年里，已经有越来越多的科学家对肺癌和RAS之间的复杂关系产生了兴趣^[26]^。众所周知，RAS在维持血压和心血管体内平衡中扮演着重要的角色^[27]^。然而，最近公布的数据^[28, 29]^表明，RAS的主要生物活性肽——AngⅡ，在肺癌的发生和发展中产生着重要影响。AngⅡ与血管内皮细胞表面的受体结合，可激活细胞内的第二信使，并通过诱导细胞入侵及组织新生血管的形成等一系列作用，实现其促进血管增生、组织修复与生长的作用。国内外多项实验^[30, 31]^均表明，血管内皮生长因子（vascular endothelial growth factor, VEGF）与肿瘤的转移相关。VEGF是体内已知的促血管生长作用最强的因子，可以刺激血管内皮细胞的有丝分裂的发生和血管的生成，增强单层内皮细胞的通透性，从而抑制肿瘤细胞的凋亡，促使肿瘤的生长、浸润及转移。与正常组织相比，VEGF在很多肿瘤组织中表达量增高，并与肿瘤的临床分期、恶性程度以及是否转移相关^[32]^。AngⅡ及其受体能够促使肿瘤血管的生成，这种作用主要是通过诱导血管内皮生长因子、血小板衍生因子、成纤维细胞生长因子和转化生长因子等完成的^[33]^。在多种类型肿瘤细胞中均发现，AngⅡ与VEGF的表达量呈正相关，两者的浓度都普遍高于正常组织细胞。有研究发现，血管紧张素通过阻断磷脂酰肌醇-3-激（phosphatidylinositol-3-kinase, PI3K/Akt）和丝裂原活化蛋白激（mitogen-activated protein kinase, MAPK）信号通路，能够抑制肺腺癌细胞系A549细胞的迁移和浸润^[34]^。由此更能提示我们，AngⅡ与肺癌的转移密切相关。曾有报道^[26]^指出，ACE2的低表达与肺癌分期有关。在目前研究中我们发现，上皮-间质细胞转化（epithelial-mesenchymal transition, EMT）在肿瘤进展和转移的形成过程中起着重要的作用，而ACE2能够通过抑制EMT从而抑制肺癌转移过程。这一发现表明，ACE2可以作为肺癌的一个潜在的治疗靶点。

### 4.2 ACE抑制剂（ACE inhibitor, ACEI）或血管紧张素受体阻滞剂（angiotensin receptor antagonist, ARB）与肺癌治疗

已有证据^[35]^表明RAS在癌症的发展和临床治疗中扮演着角色，RAS抑制剂如ACEIs和ARBs能够降低癌症患病率。已有研究^[35]^报道RAS各组成部分在各种癌症细胞和组织中表达，并且临床研究表明RAS系统失调与癌症的侵袭和转移有关。非小细胞肺癌（non-small cell lung cancer, NSCLC）患者中接受ACEIs或ARBs治疗的患者比未接受此种治疗的患者，能够获得更长的中位生存期^[35]^。在NSCLC患者治疗中，卡托普利或坎地沙坦的应用，能够分别减少肿瘤体积和淋巴结转移。ARBs药物发挥作用主要通过抑制AT1R，从而对肿瘤生长产生抑制作用^[36]^。埃克替尼治疗期间使用ARBs可能会延长转移性NSCLC患者的总生存期^[37]^。在NSCLC患者接受一线以铂为基础的治疗方案的一项回顾性研究表明，ACEI或ARB治疗可能延长生存期，但生存优势不可能简单地确定为某些风险因素，故目前尚未明确。在过去几年中的许多研究表明，ACE多态性可能与癌症的风险密切相关，包括乳腺癌、胃癌、前列腺癌。此外，一些研究还发现ACE多态性与肺癌相关，表明它可能作为肺癌的危险因素。对于恶性肿瘤与ACEI和其他抗高血压药物之间的关系，最初的研究大约在十年前。第一项研究是对1, 559例应用ACEI超过15年的高血压患者，罹患癌症的风险进行回顾性分析。在该项研究中，与对照组相比，癌症的相对风险下降了28%，在女性患者中甚至达到37%^[38, 39]^。由此可见，ACEIs和ARBs有可能成为肺癌的新型治疗药物。

综上所述，AngⅡ及其受体在肿瘤的发生、发展及其血管生成中起着重要的作用，ACEI和AT1R拮抗剂通过抑制AngⅡ的产生或者阻滞AngⅡ的作用，在抑制肿瘤的进展、转移和血管生成等方面都起着有益的作用。在对AngⅡ及其受体与肿瘤关系的研究基础上，有研究者已使用现有的药物（ACEI和AT1R拮抗剂）对肿瘤治疗进行了尝试，并取得一定的疗效。与此同时，很多针对AT1R或RAS的抗肿瘤药物正在开发和研究中。AT1R为肿瘤治疗开辟了新的药物靶点，随着对AT1R研究的不断深入，更有效的抗癌药物将被开发出来。
